# Harnessing preclinical models for the interrogation of ovarian cancer

**DOI:** 10.1186/s13046-022-02486-z

**Published:** 2022-09-16

**Authors:** Tianyu Qin, Junpeng Fan, Funian Lu, Li Zhang, Chen Liu, Qiyue Xiong, Yang Zhao, Gang Chen, Chaoyang Sun

**Affiliations:** 1 Department of Obstetrics and Gynecology, Tongji Hospital, Tongji Medical College, Huazhong University of Science and Technology, Research building of Tongji Hospital, No.2 Jiankang Avenue, Caidian Street, Caidian District, Wuhan, 430030 China; 2 Cancer Biology Research Center, Tongji Hospital, Tongji Medical College, Huazhong University of Science and Technology, Wuhan, 430030 China; 3 Department of Obstetrics and Gynecology, Shanghai General Hospital, Shanghai, 200000 China; 4 Department of Plastic Cosmetic and Burn Surgery, Taihe Hospital, Hubei University of Medicine, Shiyan, 442008 China

**Keywords:** Ovarian cancer, Preclinical models, Patient-derived xenograft, Patient-derived organoids, Genetically engineered mouse models, Personalised medicine

## Abstract

Ovarian cancer (OC) is a heterogeneous malignancy with various etiology, histopathology, and biological feature. Despite accumulating understanding of OC in the post-genomic era, the preclinical knowledge still undergoes limited translation from bench to beside, and the prognosis of ovarian cancer has remained dismal over the past 30 years. Henceforth, reliable preclinical model systems are warranted to bridge the gap between laboratory experiments and clinical practice. In this review, we discuss the status quo of ovarian cancer preclinical models which includes conventional cell line models, patient-derived xenografts (PDXs), patient-derived organoids (PDOs), patient-derived explants (PDEs), and genetically engineered mouse models (GEMMs). Each model has its own strengths and drawbacks. We focus on the potentials and challenges of using these valuable tools, either alone or in combination, to interrogate critical issues with OC.

## Background

Ovarian cancer incorporates multiple malignancies with a variety of etiology, histopathology, and biological feature, including those derived from epithelial, germ cell, sex cord stromal, and metastatic lesions [1]. On a global scale, OC causes 152,000 deaths among 239,000 new cases annually, making OC the second leading cause of cancer-related death in women [2]. Most OC patients were diagnosed at an advanced stage with extensive peritoneal metastasis, yet symptoms are rather vague [3, 4]. The dismal prognosis, limited treatment options, and high recurrence rate followed by stout resistance together render OC the most fatal gynecologic tumor, and the treatment of OC an ongoing challenge.

The current standard of care for OC is primary debulking surgery followed by platinum-based chemotherapy [5, 6]. Neoadjuvant chemotherapy might be considered for non-operable patients who are intolerant of surgery or deemed unlikely to reach a cytoreduction to no gross residual disease (R0) [7]. The advent of poly ADP-ribose polymerase (PARP) inhibitors, to a certain extent, revolutionized the treatment of patients with BRCA1/2 mutations. This is based on a theory named “synergetic lethality”, which was first described in 1946 [8]. Theoretically, dual inactivation of BRCA and PARP results in replication catastrophe, leading to the inevitable death of cancer cells. However, when it comes to treatment in a clinical setting, PARPi can lose its magic once resistance occurs, not to mention that the predominant beneficiary group carrying BRCA1/2 mutation only accounts for 15% of OC patients.

**Fig. 1:**
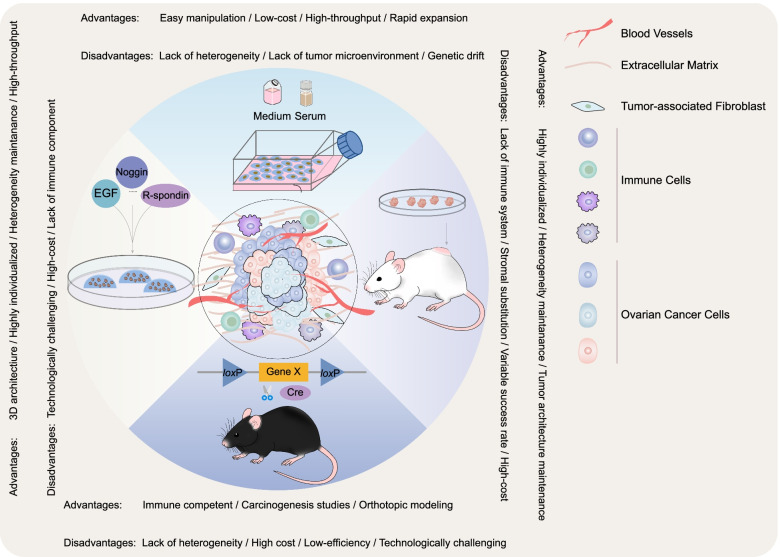
Schematic representation of the most commonly used OC preclinical models. Both advantages and disadvantages are summarized for each model

Personalized medicine allowed advances in the exploitation of alternative strategies to tackle the aforementioned bottlenecks, yet the biological insufficiency of OC pre-clinical models to fully recapitulate the complexity of OC, to a certain extent, restraints the understanding and resolving of this heterogeneous disease [9]. A solid pre-clinical model should mirror the morphological and biological characteristics of the corresponding tumor of origin to the full extent. Historically, ever since the establishment of HELA cells, 2D cell line models have long been the mainstay of experimental cancer research [10]. Immortalized cancer cells cultured in artificial FBS-based medium boosted the exploration of cancer biology, somehow, they failed to bridge the gap between laboratory experiments and clinical trials. Abortions in phase III trials were mainly due to inadequate efficacy, which underlines the discrepancy between conventional cell lines and individual patients [11–13]. In response to this intractable situation, generations of pre-clinical models have been developed, among which patient-derived xenografts, organoids, tumor explants, and genetically engineered mouse models emerged as complementary tools in cancer research and drug screening (Fig. 1). In this review, we discuss the current status of OC models, and the strengths and drawbacks of each platform, casting light on the potentials and challenges of using these tools to unravel the biological fingerprints and vulnerabilities of OC.

## Conventional cell lines

Conventional cell line models, ever since the establishment of the HELA cell line, had long been acknowledged to profoundly facilitate the understanding of tumor initiation, progression, metastasis, and drug discovery [10]. Well-established cell line models recapitulate the hallmarks of parental tumors in a way that can mimic the molecular aberrations and vital biological events. As regards to OC, there have been approximately 100 cell lines publicly available, most of which, however, lack proper annotations at histological, cellular, and molecular levels [14]. Based on transcriptomic data, Barnes BM and colleagues clustered 44 OC cell lines into 5 transcriptionally different groups, highly corresponding to the 5 major histological subtypes of OC [15]. It is worthwhile to mention that a bonafide list of reliable cell line models for distinct human pathology is needed to guide type-specific OC research [16, 17]. Currently, compiling works have been devoted to the establishment of OC cell line models with defined pathology and well-characterized phenotypic and genomic profiles. The team of Kreuzinger C innovatively established reliable high-grade serous OC (HGSOC) cell lines from clinical OC samples, while providing a detailed background in clinical parameters, chemosensitivity status, and molecular alterations. And in a further step, a total of 34 established cell lines were used to decode potential therapeutic targets specific for HGSOC [18, 19]. In addition, cell lines for ovarian clear cell carcinoma (CCC) [20], low-grade serous ovarian carcinoma (LGSOC) [21], endometrioid adenocarcinoma (EMC) [22], and other pathology were cultured and introduced by researchers worldwide.

Notably, the OC cell line toolbox is further expanded by the development of drug-resistant cells and syngeneic mouse cell lines as well. A fundamental pain point in OC treatment is the stout resistance to chemotherapies, which calls for in vitro drug-resistant models to help understand the underlying mechanism. Methodologically speaking, one option is to derive resistant models from clinically relevant samples [23, 24], another is to establish isogenic drug-resistant cell lines, usually through stepwise sequential exposure to increased concentration of drugs [25]. For example, the carboplatin-resistant A2780 cells [26], taxol-resistant OC3/TAX300 cells [27]. Interestingly, comparisons between isogenic drug-resistant cell lines and their background counterpart may serve as the lens to look into the trivia during the development of OC drug resistance [28, 29]. As for syngeneic murine cell lines, ID8 is the most widely used and also the most extensively characterized model for epithelial OC. The ability of ID8 cells to form sporadic lesions in the peritoneal cavity of immune-competent mice closely mimics the biological events in stage III and/or IV OC in patients [30]. Henceforth, syngeneic murine cell lines are still representable and the most convenient models to use for the study in the organism with full immunity and comprehensive stroma components.

OC cell line model, as a fundamental tool, remains to be the cornerstone in cancer models for its easy manipulation, low cost, and short doubling time [31]. The US National Cancer Institute (NCI) 60 human tumor cell lines representing 9 distinct tumor types (including ovarian cancer) were assembled in the late 1980s under a disease-oriented concept, aiming at identifying the anti-tumor effect of compounds in particular tumor types [32, 33]. Moving beyond its original intention, the NCI 60 panel, combined with high-throughput technology, served as a pipeline in the field of drug screening [34]. Among the 60 extensively characterized cell lines, IGR-OV1, OVCAR-3, OVCAR-4, OVCAR-5, OVCAR-8, and NCI/ADR-RES are considered to be the 6 most representative OC cell lines to undergo numerous validations of promising small molecule compound [35]. Other large-scale datasets including The Cancer Cell Line Encyclopedia (CCLE) [36], the Cancer Genome Project (CGP) [37], and Cancer Therapeutic Response Portal (CTRP) facilitate the in-depth study of drug response across cell lines.

Established cell lines are and will continue to be of paramount significance in every aspect of OC research. It is now beyond doubt that cell lines facilitated the study of cellular dependency in high-throughput pharmacological interrogation and genetic screening. Bulk of cell line studies on the genome-wide level revealed the genetic alterations related to drug sensitivity and resistance. Papp et al. integrated genomic, epigenomic, and expression analyses to shed light on the molecular abnormalities in an ovarian cancer cell line panel comprised of 45 OC cell lines and revealed unique molecular dependencies of several targeted therapies [38]. Garnett MJ et al. conducted systematic pharmacogenomic profiling in a pan-cancer cohort including 17 OC cell lines to provide an extensive view of the genomics underlying drug sensitivity. Using cell line models, large-scale loss-of-function screens such as siRNA, shRNA, and CRISPR-Cas9 library yielded new insights on identifying “driver” mutations among the “passenger” ones [39–42]. Mengwasser KE and colleagues conducted shRNA and CRISPR screening with DNA-repair-based libraries on 2 pairs of BRCA2 isogenic cell lines, including ovarian PEO1 B2MUT cells, revealing FEN1 and APEX2 as BRCA2 synthetic lethal targets, which could possibly be utilized in future treatment of BRCA-deficient populations [43]. In addition, the examples of utilizing OC cell line models to predict drug efficacy and explore underlying molecular mechanisms in OC initiation, progression, metastasis, and response to clinical and novel therapies are far too numerous to be detailed. Experimental technique evolving cell line models are well-developed and still undergoing a continuous boom. In vivo tumorigenicity of some cell lines endowed them with the utility to form xenografts for the study of tumor initiation and metastasis as well as in vivo drug response in animal experiments [44, 45].

Though historically served as the main force in cancer research models, conventional cell lines are continuously questioned in their fidelity and clinical relevance [46–50]. Serial analysis of gene expression (SAGE) database identified distinct gene clusters overexpressed in cell lines and solid tumors respectively, which might give rise to the deviations of cell lines in respect of chemotherapy resistance [47]. Comparison between OC cell lines and clinical samples revealed that cell line models failed to capture clinical MDR gene expression patterns with their high selectivity in the expression of genes associated with MDR [48]. A research comprised of 41 OC cell lines indicated that selective pressure against BRCA1/2 mutations during adaption to 2D culture might contribute to a lower incidence of BRCA1/2 deleterious mutations than the population-level incidence [51]. Another comparative study looked at the CNVs, mutations, and mRNA expression profiles between commonly used OC cell lines and HGSOC samples and revealed pronounced gaps in molecular profiles. Startlingly, the most popular OC cell lines such as SK-OV-3, A2780, OVCAR-3, CAOV3, and IGROV1 showed low correlation value with individual tumors while rarely applied cell lines such as KURAMOCHI, OVSAHO stood out as “good cell lines” [50]. However, being a representative cell line does not necessarily mean that it can successfully and efficiently form xenografts in vivo, again restraining the utility of cell line models [44, 52].

As the genomic fingerprints, even druggable targets might be lost over extensive passages in vitro, the utilization of this conventional model is fairly discouraged [48]. Notably, given the random nature of the incidence of genomic drift, it’s possible for identical cell lines to differ even among laboratories. Clearly, OC cell lines commonly used in published studies do not stand for the full complexity of OC. To summarize, conventional OC cell line models, by their very nature, cannot afford the rapid expansion of precision medicine alone.

## Patient-derived xenografts

In 1953, Toolan HW made the first systematic report on human tumors growing in cortisone-treated rats. Thereby, the PDX model began to take shape [53]. Although basic concepts and methodologies began to emerge, it was not until the introduction of immunodeficient nude mice that PDX had its first major breakthrough [54]. Bosma, G.C. introduced CB17-SCID mice deficient in immune functions mediated by T and B lymphocytes [55], followed by NOD-SCID mice (Jackson Lab) with abrogated NK cells [56] and NOD-SCID IL2rg null (NSG) mice with no functional T, B and NK cells [57]. Mouse strains mentioned above improved the success rate of transplantation, optimized the methodology, and provided researchers with more options [55–58]. Specific recipient strain can therefore be applied depending on the specific research purpose. The very first report on successful transplantation and passaging of OC tissue into nude mice could date back to 1977 when Davy M described a poorly differentiated ovarian adenocarcinoma PDX and confirmed its correspondence with the donor patient in drug response [59].

Up till now, compiling works have been devoted to the establishment, characterization and utilization of OC PDX (Table 1). To establish a PDX, fresh OC tissues obtained from surgery, biopsy, and ascites fluid were directly transplanted into immunodeficient mice orthotopically, subcutaneously, or intraperitoneally after simple initial processing. The inoculum can be tissue mass or cell suspension, either alone or coated with upholders such as Matrigel or human fibroblasts [74]. Orthotopic engraftment recapitulates the favorable anatomic microenvironment of the original tumor [75, 76]. Intraperitoneal dissemination and ascites, thereafter, can be formed based on the intrinsic metastasis mechanism of OC [77]. Subcutaneous engraftment, by contrast, is the most common choice for the convenience of observation and monitoring of tumor volume using a caliper [74]. Intraperitoneal transplantation often results in metastatic lesions in the peritoneum, omentum, pelvic and abdominal organs [64]. Albeit better resemblance in terms of biological behaviors, both orthotopic and intraperitoneal engraftment require non-invasive imaging techniques which are relatively time- and resource-consuming. A complementary evaluation method adopted by Joyce F Liu et al. is to monitor surrogate biomarkers such as CA125 and human LINE-1 [64]. Glaser G et al. came up with a physical-exam-based scoring method which was reported to share a high correlation with tumor weight at necropsy [78]. Additionally, the subrenal capsule transplantation offered another option based on a previous report that this highly vascularized site yielded a high take rate [66, 71, 79], however, this was not observed in several other studies [80].

**Table 1:** Key information and opinions in recent OC PDX research

Patients’ material	Mice strain	Grafting site	Number of models	Grafting rate	Histology	Therapy	Genetical profiling	Original findings and opinions	Reference
Tumor tissue	Nude	SQ	61	46.92%	SOC, CCC, EMC, MOC, MMMT, Brenner	NI	YES	1). engraftment rate of OC PDX was correlated with patients’ prognosis. 2). Differentially expressed genes selected according to PDX engraftment status could be a prognosis marker of CCC patients.	Shin, Ha-Yeon et al. [60]
Tumor tissue	NOD/SCID, NRG, NSG	SQ	33	76.74%	HGSOC	Cisplatin and/or paclitaxel	YES	1). PDXs remained stable in histological and genetic features throughout propagation. 2). HGSOC PDX models faithfully recapitulated the chemotherapy response of corresponding patients. 3). Development of HGSOC PDX that can be visualized by bioluminescence imaging.	Cybula, Magdalena et al. [61]
Tumor tissue	NPI	SQ	92	58.23%	HGSOC, LGSOC, EMC, CCC, MOC	Paclitaxel + carboplatin/ cisplatin, carboplatin + doxorubicin	YES	1). Despite certain deviations in transcriptomic level, OC PDXs retained the histology, protein expression, and genetic alteration of parental tumors 2). OC PDX showed significant similarity with patients in chemotherapy response.	Chen, Jiayu et al. [62]
Tumor cell suspension	NOD/SCID, NSG	MFP	38	NI	HGSOC	Carboplatin	YES	1). OC PDXs showed similar sensitivity to carboplatin as the patients’ tumor. 2). OC PDXs recapitulated the diversity of genomic alterations in HGSOC. 3). OC PDXs represented all HGSOC subtypes except for the immunoreactive group.	Cybulska, Paulina et al. [63]
Ascites, pleural effusions	Nude, NSG	IP	14	14.89%	HGSOC, ADENO, Mixed	Carboplatin and/or paclitaxel	YES	1). Histologic and molecular features were preserved through PDX passaging and post-luciferization. 2). PDX models responded to first-line chemotherapy in a way reflective of the clinical features of OC. 3). Generation of PDX models with malignant ascites and pleural effusions may better reflect recurrent treatment-resistant OC.	Liu, Joyce F et al. [64]
Tumor tissue	NSG	OTP	37	92.50%	BRCA^mut^ HGSOC	ATR/CHK1 inhibitor, PARP inhibitor	YES	1). Establishment of HR-deficient HGSOC PDX models. 2). OC PDXs were suitable models for preclinical study of chemotherapies and targeted therapies based on RPPA identification	George, Erin et al. [65]
Tumor tissue	Nude	SRC	22	48.89%	SOC, CCC	Paclitaxel + carboplatin, EGFR inhibitor	YES	1). Patients with successfully engrafted tumor had inferior OS. 2). Chemotherapy response of PDXs was concordant with that of patients. 3). Erlotinib significantly decreased the tumor weight of an CCC PDX in preclinical experiments.	Heo, Eun Jin et al. [66]
Tumor tissue	NOD/SCID, NSG	SQ, OTP	9	NI	HGSOC	NI	YES	1). OC PDXs maintained similar histologies, cellular compositions and oncogenic markers of original tumor. 2). Steroid hormone receptors loss and immunoresponsive genes alteration were obseved in PDX tumors.	Dong, Ruifen et al. [67]
Tumor tissue, ascites	Nude	SQ, IP, OTP	34	24.64%	SOC, EMC, CCC, MOC, Mixed, Brenner, others	Paclitaxel + cisplatin	YES	1). OC PDXs were histologically similar to the corresponding patient tumor and comprised all the major ovarian cancer subtypes. 2). Othotopic transplantation resulted in peritoneal tumor dessemination and ascites. 3). Drug response of OC PDXs resembled corresponding patients.	Ricci, Francesca et al. [68]
Tumor tissue	NOD/SCID	SQ, OTP	10	83.33%	HGSOC	Cisplatin	YES	1). HGSOC PDX models could inherit BRCA mutation and oncogene overexperssion in original tumor. 2). OC PDXs could be used for better design of future clinical trials.	Topp, Monique D et al. [69]
Tumor cell suspension	NOD/SCID	IP	168	74%	SOC, EMC, CCC, MOC, Mixed, Others	Carboplatin /paclitaxel	YES	1). OC PDX retained key clinical and molecular features of primary tumor, demonstrating considerable diversity. 2). OC PDX biobank can serve as accurate surrogates for OC patients for individualized therapy development.	Weroha, S John et al. [70]
Tumor tissue	NOD/SCID	SRC	11	96%	SOC, MOC, GCT	NI	NO	1). SRC xenografts and donor tissues showed highly similar histopathological features. 2). Subrenal capsule implantation yeilded achievable, consistently high enngraftment rate.	Lee, Cheng-Han et al. [71]
Tumor tissue, ascites, pleural effusions	Nude	SQ, IP	15 for IP 18 for SP	28% for IP 30% for SP	SOC, ADENO, EMC, CCC, MMMT	NI	NO	1). Overexpression of mutant P53 tended to influence the tumorigeneity of OC. 2). OC PDX panels are usful models for cancer biology and therapeutic studies.	Verschraegen, Claire F et al. [72]
Ascites	Nude	SQ, IP	4	20%	HGSOC	Cisplatin, adriamycin and cyclophosphamide	NO	1). The histology of xenografts remained stable. 2). Genetic content in PDX was not markedly different from that of original tumor, with minimal variations through passages. 3). Heterogeneity of OC in chemotherapy response was reserved in xenografts.	Massazza, G et al. [73]

*SQ* Subcutaneous, *MFP* Mammary fat pad, *IP* Intraperitoneal, *OTP* Orthotopic, *SRC* Subrenal capsule, *SOC* Serous ovarian carcinoma, *CCC* Clear cell carcinoma, *EMC* Endometrioid carcinoma, *MOC* Mucinous carcinoma, *MMMT* Malignant Müllerian mixed tumor, *HGSOC* High-grade serous ovarian carcinoma, *LGSOC* Low-grade serous ovarian carcinoma, *ADENO* adenocarcinoma, *GCT* Granulosa cell tumor, *RPPA* Reverse phase protein array, *OS* Overall survival, *EGFR* Epidermal growth factor receptor, *NI* Not informed, *ATR* Ataxia telangiectasia and rad3, *CHK* Checkpoint kinase 1, *PARP* Poly ADP-ribose polymerase

The success rates of OC PDX implantation ranged from 25% [68] to more than 95% [71] according to previous reports. The possible confounding factors are as follows. 1) Pathological type, stage, and grade of the parental tumor. In common sense, samples with more aggressive pathological features and more advanced stages are better able to be engrafted than indolent tumors [81]. 2) The quality of the tissue grafts. Tumor tissues are required to be rapidly transferred from the operating room to the experimental center and a proper medium is needed during the transfer process to ensure viability. Moreover, the segmentation of the specimen, tumor/necrosis percentage of inoculum also play a decisive role. 3) Type of the tissue implanted. The material can be solid tissue chunks or dissociated cell suspension. Metastatic lesions are reported to have a higher take rate than the primary lesion [82]. 4) The strain of recipient mice, which has been described previously. 5) The implantation site. Dobbin et al. evaluated the tumorigenicity of OC in subcutaneous (SQ), mammary fat pat (MFP), intraperitoneal (IP), subrenal capsule (SRC) site and the engraftment rates are 85.3, 63.64, 22.2, and 8.3% respectively [80]. Further systematic comparative studies focusing on improving OC engraftment rate are warranted.

As a heterogeneous disease, the research of OC requires preclinical models that are personalized to solve individual issues, and OC cell lines, as discussed previously, are clearly not up to it. Recently, the NCI decided to take up with PDXs for drug screening as a substitution for the NCI-60 cell line panel, considering that PDX better mimics the human tumor [46]. In fact, PDX models are competent to capture therapeutic candidates that are missing in cell line screening [83]. Numerous studies have demonstrated that the histological structure and genomic signatures (including the mutation profile, CNV, MSI, and STR) are faithfully preserved in murine models and remain stable even after several sequential passages between recipient mice [74]. Liu Y. et al. examined the molecular fidelity of OC PDX versus primary ovarian tumors at the mRNA level and devised a bioinformatic pipeline to separate the murine component transcriptome confounders [84]. Consequently, individual PDX can serve as an avatar model for donor patients, retaining the biological uniqueness to undergo laboratory and clinical interrogation. By expanding PDX through mouse-to-mouse passages, a large cohort of tumor-bearing mice testable can therefore be assembled to receive multiple trials and determine the best regimen for OC. Provided that an OC PDX bank comprising a variety of patients, it can simulate clinical trials based on molecular pathological and molecular characteristics of a real OC patient population. Such concept of “xeno-trials” motivated researchers and organizations to set up international collaborations, for example, the EurOPDX and PDXNET consortium [74, 85]. By integrating worldwide PDX panels together with corresponding clinical data, genomic information, and drug responsiveness, authoritative resources can be achieved to facilitate precision medicine [58].

The advantages of the OC PDX model have evoked promises to tackle crucial issues in OC research. Via serial passaging, the original tumor tissue can be expanded on host mice and harvested to undergo various manipulations. Tissue bio-banked together with tumor-bearing mice, which is a living bio-bank itself, constitute a resourceful OC library [86]. The highlight of PDX has always been to validate the efficacy of various regimens. Parmar K. et al. assessed the activity of prexasertib and its combination with olaparib in 14 clinically characterized PDXs [87]. Cornelison R. et al. utilized PDX with or without chemotherapy to examine the potential of targeting ribosomal machinery in OC [88]. In general, compared with traditional cell lines and cell line-derived xenografts (CDX), OC PDX serves as a better and more convincing pre-clinical model in testing drug response of chemo- and targeted therapy. Consequently, the application of PDX is gaining momentum and has gradually become mainstream in OC research. George E et al. introduced a BRCA-deficient OC PDX platform to test novel targeted therapies [65]. Moving beyond the laboratory field, Colon-Otero G. et al. made the first attempt at PDX co-clinical trial in OC [89]. Unfortunately, this study only attempted to establish PDX with biopsy samples from tested patients, leaving a gap in the drug response of the corresponding tumor-bearing xeno-patients. Pioneering works incorporating PDX cohorts in clinical trials have been done in breast cancer, lung cancer, soft tissue sarcoma et al. [90–94] and we expect further exploration in OC to come. Apart from pre-clinical drug testing, PDX is a suitable tool for biomarker identification and validation. One example comes from clear cell ovarian carcinoma PDX to address the mechanism of carboplatin resistance and the study identified APOBEC3B as a new biomarker for intervention [95]. Palmer AC et al. analyzed the genetic profile of OC PDX models exposed to 21 monotherapies and reported that nearly 90% of models responded to at least one biomarker-guided therapy [96].

Nevertheless, there are certain inherent weaknesses of PDX models that cannot be ignored in OC research. Immunodeficient recipient strains lack key immune components. On the one hand, it avoids host versus graft rejection and enables successful engraftment. On the other hand, it severely restrains the utility of PDX in the exploitation of immune-related therapies, which is a key topic in OC research [74]. In order to solve this issue, humanized mice in which human immune components are introduced into immunocompromised mice have been developed. The immune reconstruction procedure may utilize human peripheral blood, tumor-infiltrating lymphocytes (TILs), or CD34^+^ human hematopoietic stem cells [97]. Transplantation of different human immune materials renders mice with different immune competencies. In addition, previous reports have confirmed that tumor stroma, though preserved in tumor fragments, is rapidly replaced by mouse counterparts even in the first generation [98]. The taking over of murine stroma altered the transcriptomic signature of the original tumor [99]. A study by the group of Blomme A has previously demonstrated that murine stroma adopts a metabolic phenotype similar to human [100], still, the discrepancies in drug response may occur to an unknown degree. Additionally, the adaptation of cancer cells to the murine microenvironment throughout passages might give priority to certain subpopulations, namely clonal selection [101, 102]. OC is known to be a type of cancer with high heterogeneity, which can be undermined by the enrichment of dominant clones in murine models. The subclone constitution is constantly altered by selection pressure. Therefore, early passages ensure better consistency and predictive value when questing biology and molecular clues [99]. Besides, PDX models are rather hard to handle on a genetic level, genetical intervention could be implemented via managing cell suspension during the passaging process. Last but not least, in view of both known and unknown contributing factors leading to successful engraftment, any PDX archives certainly do not represent the full spectrum of OC.

## Patient-derived organoids

In 2009, Hans Clevers and colleagues released a landmark study which documented the initiation of crypt-villus organoids by single sorted Lgr5^+^ stem cells [103]. This ground-breaking milestone kick-started a brand-new area in stem cell research as well as in 3D culture technology, followed by mountainous study devoted to the development, optimization, and broader utilization of organoid models [104–106]. Naturally, organoid was thought to hold great potential to prompt preclinical OC research based on the fact that the delicate 3D structure, to a large extent, carries the phenotypic and genomic characteristics of original tumors, and in the meanwhile preserves the inter- and intra-patient heterogeneity which are major topics of interest in the study of OC [107]. By mechanical or enzymatic digestion, the tumor tissue is embedded in the in vitro 3D matrix, whereby a homoeostatic environment is mimicked by adding growth factors and small molecule inhibitors cocktail. The artificial niche environment allows stem cells to self-organize into 3D structures, self-renew, and multi-differentiate to keep viability and integrity. These features endowed the model with the merits of biological stability through extensive passaging [108]. So far, organoid models of gastrointestinal cancers [109], glioblastoma [110], colorectal cancer [111], pancreatic cancer [112], prostate cancer [113] have been built, setting paradigms for their respective cancer research fields. With regard to OC, Hill et al. innovatively reported a short-term cultured OC platform to identify targetable DNA damage repair defects in parent tumors [114]. The first major breakthrough in expandable OC organoid came from Kopper O’s group. In this pioneering study, an OC organoid platform was built, which enables long-term expansion and genetic manipulation. And the organoid models of various OC subtypes were demonstrated to share high similarity with the corresponding tumor at histological and genomic levels [115]. Subsequent works mainly focused on the fidelity and utility of this cutting-edge technology in the OC research field (Table 2).

**Table 2:** Key information and opinions in recent OC PDO research

Patients’ material	Number of models	Success rate	Extracellular matrix	Expansion	Histology	Therapy	Genetical profiling	Original findings and opinions	Reference
Tumor tissue	13	85%	Matrigel	≥ 3 passages	HGSOC, EMC, CCC, SBT, KruKenberg	PARP inhibitors	YES	1). OC PDOs’ response to first-line chemotherapy correlated with clinical response. 2). PDOs resembled parental tumors with an average overlap of 91.5% of SNVs and SVs. 3). PDO model is capable of evaluating PARPi sensitivity, exploring resistant mechanisms, and identifying effective combination strategies.	Tao, Mengyu et al. [116]
Human iPSC	3	NI	Matrigel	NI	STIC	PARP inhibitors	YES	1). BRCA1^mut^ patient iPSC lines can differentiate into FTE. 2). BRCA1^mut^ fallopian tubes recapitulate OC tumorigenesis in vitro/vivo. 3). BRCA1^mut^ fallopian tubes provided model to predict disease severity. 4). BRCA1^mut^ fallopian tube organoids provided platform to study drug efficacy.	Yucer, Nur et al. [117]
Tumor tissue, Ascites	21	82.7%^a^	Matrigel	≥ 2 passages	HGSOC, LGSOC, CCC, EMC	Trastuzumab, gemcitabine, bevacizumab, topptecan, paclitaxel, carboplatin	NO	1). The establishment of gynecological cancer PDOs was feasible and helpful to studying the impact of drugs in a clinically meaningful time window. 2). Neoadjuvant therapy negatively affected the success rate of PDO generation.	Bi, Jianling et al. [118]
Tumor tissue	25	80%	Matrigel	2 ~ 5 passages	HGSOC, EMC, CCC, MOC, MBT, others	23 FDA-approved drugs	YES	1). Organoids captured subtype-specific characteristics of OC and replicated the mutational profile of the primary tumors. 2). Using PDO was a reliable strategy for drug testing.	Nanki, Yoshiko et al. [119]
Tumor tissue, Ascites	36	NI	Matrigel	NI	HGSOC, LGSOC, EMC, CCC, S/MBT, MOC	16 chemo- and targeted therapies	YES	1). OC PDOs could serve as drug screening models in OC research. 2). OC PDOs recapitulated patients’ responses to carboplatin and paclitaxel. 3). OC PDOs displayed inter- and intrapatient drug response heterogeneity. 4). OC PDO drug response heterogeneity can be partially explained by genetic aberrations.	de Witte, Chris Jenske et al. [120]
Ascites, pleural effusions	14	NI	Cultrex BME	Short-term for ≥6 days	HGSOC	12 chemo- and targeted therapies	YES	1). A short-term PDO culture can be applied to study drug susceptibilities for individual patient. 2). Drug screen on PDO could be beneficial for treatment-exhausted subgroup.	Chen, Hui et al. [121]
Tumor tissue	12	44%	Cultrex BME	1 ~ 2 passages	HGSOC, LGSOC, CCC, MOC	Paclitaxel, cisplatin, doxorubicin, gemcitabine	YES	1). Established organoids demonstrated parental tumor-dependent morphology and biology, retained parental tumor’s marker expression and mutational landscape. 2). Organoids exhibited tumor-specific sensitivity to clinical chemotherapies.	Maenhoudt, Nina et al. [122]
Tumor tissue	15	30%	Matrigel	6 ~ 26 passages	HGSOC	Carboplatin	YES	1). OC PDOs matched the mutational and phenotypic profiles of original tumor. 2). Wnt pathway activation led to growth inhibition of OC PDOs and active BMP signaling is almost always required for the generation of HGSOC organoids.	Hoffmann, Karen et al. [123]
Tumor tissue	9	60%	Matrigel	NI	EMC, Brenner, HGSOC, MOC, SBT	Paclitaxel, cisplatin	YES	1). PDOs retained both histological and molecular features and intra-tumoral heterogeneity of parental tumors. 2). Organoids facilitated the preclinical studies on both inter- and intra-tumor heterogeneity.	Maru, Yoshiaki et al. [124]
Tumor tissue	56	65%	Matrigel	3 ~ 31 passages	HGSOC, LGSOC, EMC, CCC, MOC, S/MBT	Paclitaxel, carboplatin, alpelisib, pictilisib, MK2206, AZD8055, niraparib, adavosertib, gemcitabine	YES	1). OC organoids recapitulate histological and molecular features of the original lesions, recapitulating intra- and interpatient heterogeneity, and can be genetically manipulated. 2). OC organoids can be used for drug-screening and capture subtype-specific responses to chemotherapy, including the development of chemoresistance in recurrent OC. 3). OC organoids can be engrafted to form corresponding PDX, enabling in vivo drug-sensitivity tests.	Kopper, Oded et al. [115]
Tumor tissue, pleural effusions	33	80–90%	Matrigel	2 passages	HGSOC, LGSOC	Carboplatin, olaparib, prexasertib, VE-822	YES	1). OC PDOs matched the parental tumors, both genetically and functionally. 2). PDOs can be used for DNA repair profiling and therapeutic sensitivity testing and provide a rapid means of evaluating targetable defects in the parent tumor, facilitating better therapeutic options.	Hill, Sarah J et al. [114]

*iPSCs* Induced pluripotent stem cells, *HGSOC* High-grade serous ovarian carcinoma, *EMC* Endometrioid carcinoma, *CCC* Clear cell carcinoma, *SBT* Serous borderline tumor, *STIC* Serous tubal intraepithelial carcinoma, *LGSOC* Low-grade serous ovarian carcinoma, *MOC* Mucinous carcinoma, *MBT* Mucinous borderline tumor, *PARP* Poly ADP-ribose polymerase, *PARPi* PARP inhibitors, *FDA* Food and drug administration (USA), *SNV* Single nucleotide variant, *SV* Structural variant, *NI* Not informed

^a^overall success rate for OC and endometrial tumor organoids

When compared with monolayer cell culture, organoid captures a more diverse repertoire that incorporates early-stage neoplastic lesions, contributing to the spectrum of cancer and precancerous models. Considerable discrepancies were reported between conventional cell lines and 3D culture systems. The underlying possibilities for this phenomenon are not only manifold but involute. Lack of cell-cell and cell-matrix interaction can be partly blamed while the diffusion rate of nutrients and metabolic waste might differ greatly in cell aggregates as well [125]. The structural difference in the culturing system brings genomic unconformity and the consequences are far more than that. Loessner D et al. reported a higher survival rate in spheroid-grown OC cells compared to their monolayer counterparts when exposed to paclitaxel treatment, which is the current first-line therapy for OC [126]. One pioneering work conducted by Jabs J’s group in exploiting OC organoid responses to clinically relevant drugs under respective genome alteration backgrounds highlighted the culture system dependency concerning cytostatic drug effect and pharmacogenomic associations [127]. With the aid of DeathPro, an originally designed automated workflow, cell death, and growth arrest rates were evaluated, and it was surprising to find drug effects clustered according to culture type to the same extent as intrinsic tumor heterogeneity [127]. Hopefully, the organoid model is endowed with the ability to uncover key information concealed by monolayer culture, demonstrating more potential and caveats in personalized drug development and biomarker validation. Apart from that, the successful establishment of cell lines is often unpredictable [72]. Generating organoid culture from normal and cancer specimens saves the trouble of getting rid of fibroblast contamination compared with cell line establishment [112].

**Fig. 2:**
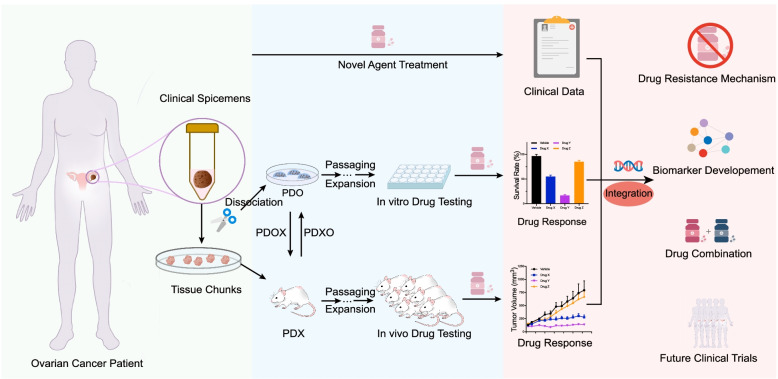
Schematic representation of co-clinical trial approach with patient-derived OC preclinical models. PDX and/or PDO models are derived from individual OC patients enrolled in a trial. Clinical data from treated patients, in vitro drug response from PDO models, and in vivo drug response from PDX models are collected and integrated with the corresponding sample to undergo comprehensive functional and genetical analysis. The clinical relevant models can therefore be exploited to facilitate the study of drug resistance mechanisms, biomarker development, and drug combination strategy, and to guide future clinical trials. PDX, patient-derived xenograft; PDO, patient-derived organoid; PDXO, PDX-derived organoid; PDOX, PDO-derived xenograft

With PDX remains to be the gold standard in the in vivo settings, PDO overcomes several major concerns with regard to PDX. When it comes to preclinical models for OC, one has to consider the expansion rate, and whether that rate provides a suitable time window for decisions to be made under the clinical conditions of OC patients. The rapid expansion capabilities of organoids set up a compatible timeline to keep pace with clinical decision-making. This clinically meaningful time window makes room for real-time co-clinical trials to guide clinical treatment (Fig. 2). Besides, unlike PDX which can only culture malignant tissues, PDO can closely mimic the normal physiology and anatomy of normal tissues by supplementation of appropriate growth factor to preserve the stem cell niche. Similarly, pre-cancerous lesions and tumor types and stages that are underrepresented in PDX modeling can be replenished by PDO [128]. For example, Kessler M et al. established stable human fallopian tube organoids and the generation of mutant organoids was further expected for the study of tumor evolution [129]. Moreover, normal tissue organoids can be used to evaluate the non-specific cytotoxicity of drug candidates, ensuring treatment safety. Bi J et al. created paired tumor and adjacent normal tissue organoids from the same patients and described minimal cell killing of several chemotherapeutic drugs at a tumor-killing dose [118]. Besides, compared with PDX which is unsuitable for high-throughput drug screening, PDO can be exploited as a complementary screening platform at a relatively low cost [130, 131]. Overall, PDO reconciled the pros and cons of traditional cell lines as in vitro models and PDX as in vivo models and bridges the gaps between laboratories and clinical trials.

In terms of applications of PDO, so far, attempts have been made on drug-screening procedures in various cancer types and systematic methodology has been recently reviewed in detail [132]. For OC, Hill SJ et al. devised a short-term HGSOC PDO platform to functionally profile DNA repair defects and further predict the therapeutic effects of drugs targeting DNA damage response (DDR) [114]. Nanki Y et al. performed drug sensitivity and resistance testing of 23 FDA-approved compounds on HGSOC PDOs and proved high concordance of the models [119]. Except for distinguishing responders and non-responders in the laboratory and preclinical drug screening, PDO can be used to capture spatially and chronologically different lesions from one exact patient [133, 134]. Considering the high recurrence rate of OC, patients might undergo multiple invasive medical interventions successively with tissues available to chronologically develop organoid culture. Ideally, the chronological model series could be used to inform clinical decisions and help to monitor the development of drug resistance in a real-time manner. Metastatic lesions of OC can be harvested and built into organoid models concurrently to verify drug response under a heterogenous background [135]. de Witte CJ et al. examined intra-patient heterogeneity and were surprised to find that organoids derived from a patient at different sites varied in monotherapy response of 31% drugs [120].

Moreover, the OC organoid model is expected to be instrumental in immunotherapy research based on its highly manipulatable nature. With immune components incorporated into the organoid culture, the co-culture system poses promising bases for immuno-oncology investigations and facilitates individualized immunotherapy screening [136, 137]. Neal et al. adopted an air-liquid interface (ALI) system to embed tumor cells with T, B, NK cells, and macrophages. The robustness of the model was proved by the accurate preservation of the parental tumor T cell receptor (TCR) spectrum [137]. Disappointingly, Immunotherapies represented by immune checkpoint blockade (ICB) agents demonstrated minimal efficacy in OC which requires a better understanding of the contributions of immune components of OC. Wan C et al. generated organoids from 12 HGSOC patients and co-cultured the models with a full component of intra-tumoral immune cells to study the mechanisms of ICBs. The application of ICBs led to decreased T cell and NK cell exhaustion and BRD1 was identified as a possible immune therapy target [138]. Other tumor microenvironment (TME) elements such as cancer-associated fibroblasts (CAF) and human blood vessel components recapitulate other aspects of the tumor microenvironment in vitro [139, 140]. For example, human umbilical vascular endothelial cells were added into OC organoid system to better mimic the early organogenesis of the human fallopian tube [141]. Additionally, the role of cancer-associated mesothelial (CAM) cells in OC was studied in a co-culture system and was proved to promote tumor chemoresistance [142].

Furthermore, organoid models are inherently amenable to genetic manipulation. Genetic aberrations can be easily introduced into culture systems by transfection and viral infection. This feature is favorable for cancer modeling and oncogene validation [143, 144]. In the field of OC research, Zhang S et al. modeled the initiation of HGSOC in mouse fallopian tube epithelial (FTE) organoids by lentivirus transduction and/or CRISPR/Cas9 mutagenesis [145]. More recently, exponential interest has been fostered in PDO co-clinical trials. Rational combining PDO, as well as PDX in clinical trials, facilitates information integration, biomarker development, and pharmacodynamic monitoring [104]. A clinical observational trial has been launched to investigate potential therapeutic for OC (NCT04555473).

Despite the great promise of PDO in OC research, translational challenges still remain. First, there are noticeable differences in the procedure of organoid establishment and passaging among different researchers. A standardized ingredients and procedure of OC organoid culture and drug response assessment criterion are further warranted. Besides, the assay methods of this 3D culture system are fairly limited and the protocols are more sophisticated and require a relatively high workload. It is gratifying that efforts have been made by experts to confront these challenges [146]. We could hold enthusiasm and expectation to explore more possibilities with OC PDO.

## Patient-derived explants

Another patient-derived model taken into consideration is the patient-derived explants (PDE), which refers to the short-term ex vivo culture of freshly obtained, surgically resected human tissue, either in chunks or cut into slices [147]. Although the methodology of PDE and PDE-related drug testing has been around for a long time, unfortunately, it has not translated into a wide range of applications and thus has not become mainstream in cancer research, particularly in OC. Limited reports have been made on the preclinical application of this model. Hence we will briefly introduce the PDE model and its application in OC research.

Technologically, the thickness of explants might affect the viability during culture and drug response considering the efficiency of nutrition diffusion and metabolites transportation. Manual slicing with surgical equipment used to be the most common method in tissue preparation yet has now been gradually replaced by mechanized methods such as the vibratome [148], which can uniform the thickness of slices. According to the report of Parajuli N et al., a slice thickness of 160 μm is optimal for tissue handling and viability [149]. There is no universal formula for PDE culture conditions, tissue-specific supplements are thought to improve the viability of different types of PDEs [150, 151]. Explants could be placed on support media such as gelatin sponges and pore membranes or could be cultured in a free-floating manner [147]. OC tissue could be cultured for about 5 days on gelatin sponges in RPMI1640 media with serum added [151]. Abreu S et al. reported OC free-floating PDE culture which reached the longevity of 30 days in DMEM media with serum addition [152]. Both studies lack supplementary additives to extend the lifetime of OC PDE models. Another technical issue is the endpoint analysis of PDE, which is critical for the evaluation of drug response. The most frequently used strategy is to measure cell viability with MTT assay after enzymatic digestion of the explant. Immunohistochemistry analysis of protein markers of cell proliferation and death is another commonly adopted method. Recently, multiple-immunofluorescence was adopted by researchers, offering innovative analytical approaches and better interpretation for PDE assays [147].

Though less adopted in OC research, the PDE model has its own unique advantages compared with PDX and PDO models. Firstly, PDE can be rapidly generated post-surgery without much technological and financial burden [153]. Besides, PDE retained the cell-cell interaction and cell polarity in a much more proper way. The existence of resident immune cells was validated by immunohistochemistry on OC PDE, showing evidence of the perseverance of tumor-infiltrating CD4^+^ and CD8^+^ T cells, macrophages and B cells [152]. Though more intuitively correlating drug response with patient pathology, the longevity of PDE severely restricted its practical applications. However, we hold the belief that by rationally combing this short-term culture with other models in OC research, PDE can assist in OC precision medicine.

## Genetically engineered mouse model

The first transgenic onco-mice generated by pronuclear injection of oncogene DNA were reported in the 1980s, initiating a novel field of genetically engineered mouse models (GEMMs) created by accurate manipulation of specific gene expression [154]. So far, the application of this sophisticated mouse model includes but is not limited to various types of cancers, helping to figure out the multistage in tumor initiation and progression, validate candidate genes and assess therapeutic efficacy [155]. Though tumor xenografts remain the most extensively applied mouse models in preclinical research, GEMMs circumvent several issues with xenografts that were discussed in the previous chapter [156]. In GEMMs, tumor cells are generated de novo in the context of a native milieu and within a whole organism. This way, the crucial tumor properties and modulators, such as immune cells and stromal elements are preserved and tumor cells can therefore co-evolve with the surrounding microenvironment [155].

Despite the success of GEMMs in other tumor types, OC GEMMs remain less than satisfactory. The putative reason is complex, yet can mainly be attributed to the paucity of prior knowledge of the origin and genetic basis of OC [157]. Above all, there are still controversies regarding the precursor of OC. On the genetic level, mutations in TP53 predominated the mutational spectrum of OC, other extensively reported alterations include RB1, EGFR, PTEN, PI3K/AKT, BRCA1/2, and KRAS et al. [158]. But how and to what extent perturbation of these genes contributes to the oncogenesis and progression of OC remains enigmatic. Efforts have been made to explore the consequence of the ablation or overexpression of genes of interest in OC GEMMs, which has been reviewed in Table 3.

**Table 3:** Key information and opinions in recent OC GEMMs research

Targeted genes	Targeting technology	Histotype	Original findings and opinions	Reference
Brca1, Tp53, Pten, Lkb1	CRISPR-Cas9	HGSOC	1). Quadruple deletion of Brca1, Tp53, Pten, and Lkb1 resulted in ovarian surface papillary tumors 4 months post-TAM. 2). Within 6 months post-TAM, widespread peritoneal metastasis formed in the Lkb1 deletion cohort, and some mice generated ascites by 7 months post-TAM. 3). Between 6 and 14 m post-TAM, the incidence of peritoneal metastasis was 96% and the incidence of ascites was 74% in the Lkb1 deletion cohort.	Teng, Katie et al. [159]
Trp53, Pten, Rb1, Cdh1	Amhr2 promoter driven Cre	LGSOC, HGSOC	1). Triple deletion of Trp53, Pten, Rb1 initiated OC development in OSE cells. 2). Additional Cdh1 ablation promoted tumor dissemination and ascites formation.	Shi, Mingxin et al [160].
Rb1, Brca1, Trp53 and/or Nf1	Ovgp1-TAM promotor driven Cre	HGSOC, MMMT	1). FTE-specific inactivation of Brca1, Trp53, Rb1, and Nf1 resulted in STICs that progressed to HGSOC, with widespread metastases in some cases. 2). Brca1, Trp53 and Pten inactivation in the oviduct resulted in STICs and HGSOCs and was associated with diffuse epithelial hyperplasia and mucinous metaplasia. 3). Tumour initiation and/or progression in mice lacking conditional Pten alleles probably require the acquisition of additional defects.	Zhai, Yali et al. [161]
Pten, Apc	Ovgp1-TAM promotor driven Cre, AdCre	EMC	1). Oviductal epithelial hyperplasia and atypia formed ~ 1 month post-TAM. 2). Well-formed oviductal EMC-like tumors formed 9–12 weeks post-TAM. 3). 10 of 15 mice had extensive OC, 4 with omentum metastases; 1 with lung metastases.	Wu, Rong et al. [162]
Arid1a, Pten;Apc	AdCre	EMC	1). Arid1a inactivation enhanced epithelial differentiation in a murine model of EMC. 2). Arid1a inactivation resulted in prolonged survival in the Apc/Pten-deficient EMC model.	Zhai, Yali et al. [163]
Pten, Kras, Trp53	Amhr2 promoter driven Cre	MOC, LGSOC, SOC	1). Trp53^R172H^ mutation promoted EOC but differently contribute to the disease in the presence or absence of the wild-type TP53 allele. 2). Ovarian tumors homozygous for Trp53^R172H^ mutation were undifferentiated and highly metastatic, exhibited minimal TP53 transactivation activity, and expressed genes with potential regulatory functions in EOC development.	Ren, Yi A et al. [164]
Apc	Pgr promotor driven Cre	EMC	1). In 87.2% of Pgr^Cre/+^; Apc^ex15lox/lox^ mice, lesions were found in the epithelium of the distal oviduct and fimbriae. 2). In 16.3% of mice, endometrioid cysts were detected. 3). In 27.9% of mice, endometrioid ovarian tumors developed.	van der Horst, Paul H et al. [165]
Trp53, Brca1, Brca2, Pten	Pax8-TET promotor driven Cre	HGSOC	1). Deletion of Brca1 or Brca2, Tp53, and Pten in FTE resulted in STIC lesions, HGSOC, and the progression to advanced stage disease with metastases. 2). GEMM tumor showed human HGSOC biomarkers and genomically correlated with TCGA data.	Perets, Ruth et al. [166]
Trp53;Rb;Brca1;Brca2	AdCre	HGSOC	1). Inactivation of RB induced surface epithelial proliferation with progression to stage I carcinoma. 2). Additional biallelic inactivation and/or missense p53 mutation in the presence or absence of Brca1/2 caused progression to stage IV disease.	Szabova, Ludmila et al. [167]
Dicer1, Pten	Amhr2 promoter driven Cre	HGSOC	1). Dicer-Pten double-knockout resulted in aggressive primary fallopian tube tumors with ascites. 2). Fallopian tube removal at early age prevented tumor formation, confirming the FTE as tumor origin.	Kim, Jaeyeon et al. [168]
Pten, Pik3ca	AdCre	SOC; GCT	1). Pik3ca mutation requires a second hit to initiate tumorigenesis in the ovary. 2). Pik3ca^H1047R^ or Pten deletion in the ovary induced serous papillary hyperplasia and cooperated to induce SOC or GCT.	Kinross, Kathryn M et al. [169]
Pten, Kras	Amhr2 promoter driven Cre	LGSOC	1). Mutant mice developed LGSOC at an early age and with 100% penetrance. 2). KRAS is a key driver of OSE transformation.	Mullany, L K et al. [170]
Trp53, Brca1, c-Myc	Retrovirals-depended Cre	SOC	1). Myc could induce malignant transformation in Brca1 and p53 deficient cells but was not sufficient for the transformation of cells deficient for either Brca1 or p53.	Xing, Deyin et al. [171]
Pten, K-ras	AdCre	EMC	1). GEMMs showed endometriosis-like lesions within the OSE but no invasive ovarian tumors up to 10 months post-infection. 2). All GEMMs developed invasive EMC as early as 7 weeks post-infection	Dinulescu, Daniela M et al. [172]
Trp53, Rb1	AdCre	EOC	1). Dual inactivation of p53 and Rb1 is sufficient for reproducible induction of ovarian epithelial carcinogenesis in mice homozygous for conditional gene alleles. 2). Ovarian neoplasms spread intraperitoneally with ascites, and metastasize to the contralateral ovary, the lung, and the liver.	Flesken-Nikitin, Andrea et al. [173]
Trp53, c-Myc, K-ras, Akt	Retroviral gene delivery	NI	1). Addition of any two of the oncogenes c-myc, K-ras, and Akt were sufficient to induce maliganant transformation in ovarian cells deficient for p53, 2). The induced ovarian tumors in mice resembled human ovarian carcinomas in their rapid progression and intraperitoneal metastatic spread.	Orsulic, Sandra et al. [174]

*HGSOC* High-grade serous ovarian carcinoma, *LGSOC* Low-grade serous ovarian carcinoma, *MMMT* Malignant Müllerian mixed tumor, *EMC* Endometrioid carcinoma, *MOC* Mucinous carcinoma, *SOC* Serous ovarian cancer, *GCT* Granulosa cell tumors, *EOC* Epithelial ovarian cancer, *TAM* Tamoxifen, *TET* Tetracycline, *STICs* Serous tubal intraepithelial carcinomas, *FTE* fallopian tube epithelium, *OSE* Ovarian surface epithelium, *NI* Not informed

Another factor that confounds the path of developing OC GEMM might be the lack of validated ovary-specific promoters to facilitate tissue or cell-specific functioning of the gene-editing system. In 2003, Connolly et al. reported the first transgenic mouse with poorly differentiated ovarian carcinoma which was developed by induced expression of the transforming region of SV40 driven by the Müllerian inhibitory substance type II receptor gene promoter (MISRII) [175]. SV40 Tag could functionally inactivate the tumor suppressor gene RB and P53, leading to malignant transformation of epithelial cells [176]. Using this method, approximately 50% of cases successfully developed into bilateral OC with peritoneal metastasis and ascites, which shared high similarity with clinical OC patients and thus were of high clinical relevance. In addition, the malignant ascites of the model were further utilized to establish a cell line model, which exhibited the key properties of epithelial OC. Notably, as a major defect in this transgenic model, the infertility of the female mice precluded the stabilization and expansion of this transgenic line [175]. In order to solve this issue, Connolly’s group generated an affected male founder, TgMISIIR-Tag-DR26, the female offspring of which would develop bilateral ovarian tumors with varying latency and similar histological characteristics of HGSOC. By backcrossing the TgMISIIR-TAg transgenic line, the authors obtained murine ovarian carcinoma (MOVCAR) cell lines from the malignant ascites of tumor-bearing C57BL/6 TgMISIIR-TAg transgenic mice [177]. Next, the advent of the Cre-loxP system helped to explore more possibilities in OC GEMMs development [157]. Regarding the mechanism of this mammalian gene-editing technology, Cre recombinase discovered from bacteriophage P1 recognizes a 34 base pair specific sequences called loxP site and meditates exact recombination between two loxP sites that flank the target gene. Apart from gene excision, the preset location and orientation of loxP sites can also mediate gene translocation and inversion [178]. Later, strategies were explored including AdCre injection into the ovarian bursa and Amhr2, Pax8, and Ovgp1-mediated Cre expression in Müllerian-derived epithelia and rendered various OC phenotypes under the manipulation of different suspicious OC driver genes [179].

The most extensive application of GEMMs lies in the study of cancer initiation and progression. A quadruple combination of perturbations including Pten, Trp53, Rb1, and/or Cdh1 was adopted by Shi M et al. using Amhr2cre/+ mice, developing invasive OC with extensive peritoneal metastasis by targeting ovarian surface epithelium [160]. The cell-of-origin of HGSOC remains controversial during the past decades. GEMMs are suitable models to decipher this critical question [180–182]. Flesken-Nikitin A et al. identified the hilum region of the mouse ovary as a previously undefined stem cell niche of the OSE and were susceptible to malignant transformation into epithelial OC. Hilum cells showed preferential transformation after conditional deletion of Trp53 and Rb1 using the Ad-Cre/LoxP system [183]. By introducing genetic abnormalities of combined RB family inactivation and Tp53 mutation in Pax8^+^ FTE and Lgr5^+^ OSE or OSE-derived organoids, Zhang S et al. confirmed that HGSOC may originate from both FTE and OSE and the biological behavior of tumor might vary between different tumor of origins [184].

Collectively, OC GEMMs still face major challenges both in development and application. This kind of model may have a relative disadvantage in mirroring the heterogeneity of OC, but as critically emphasized, GEMM bears an irreplaceable value in the study of carcinogenesis and the cross-talk between immune and stromal components and cancer cells.

## Conclusions

The past few years have witnessed accumulating knowledge concerning the tumorigenesis, progression, and evolution of ovarian cancer, which can be largely attributed to the development of a plethora of faithful preclinical OC models. It is now clear that OC is hallmarked by a high degree of inter- and intra-patient heterogeneity. By phenocopying the original tumor and/or expanding vital patient-derived tissues, tumor experimental models allow in-depth preclinical assessment of drug candidates and identification of tumor biomarkers at an individual level. The development and optimization of OC models are still actively ongoing, with the overall goal of better management and even the cure of OC.

Given the inherent strengths and drawbacks of each model, it is imperative to be aware that one single model alone is definitely not competent to cover all OC research. Wise selection and rational combination of OC models are instrumental in solving the pain points in respect of OC. GEMMs are naturally suitable models for studying the cell of origin of OC and can be complemented by the newly developed OC organoid model. For the study of tumor invasion and metastasis, GEMMs and orthotopic PDX models can be utilized to mimic the biological process, otherwise, cell line and organoid models could be used to exploit the underlying mechanism. As for capturing and deciphering the heterogeneity and clonal evolution of OC, the highly individualized PDX, PDO, and PDE models stood out as edged tools. Ulteriorly, the patient-derived personalized models could be applied to drug development and repurposing, serving as “avatar models” for individual patients and further facilitating patient stratification, drug response assessment, and biomarker development in a clinical setting.

To conclude, research in the post-genomic era yielded brand new insights into the biological and genetic fingerprints of OC. Henceforth, robust tumor models are required to validate the insights and distinguish those of value and therefore targetable. Conventional cell lines, PDXs, PDOs, PDEs, and GEMMs are all historically indispensable models for OC research. Especially, to realize the full translation from bench to beside, the new generation of patient-derived models will undoubtedly grow to be the mainstream in precision medicine. Future OC research should flexibly adopt suitable experimental models for various applications.

## Data Availability

Not applicable.

## Abbreviations

OC: Ovarian cancer
PDX: Patient-derived xenograft
PDO: Patient-derived organoid
PDE: Patient-derived explant
GEMMs: Genetically engineered mouse models
PARP: Poly ADP-ribose polymerase
PARPi: PARP inhibitor
BRCA: Breast cancer susceptibility gene
FBS: Fetal bovine serum
HGSOC: High-grade serous ovarian carcinoma
CCC: Clear cell carcinoma
LGSOC: Low-grade serous ovarian carcinoma
EMC: Endometrioid adenocarcinoma
NCI: The National Cancer Institute
CCLE: The Cancer Cell Line Encyclopedia
CGP: The Cancer Genome Project
CTRP: The Cancer Therapeutic Response Portal
CRISPR: Clustered regularly interspersed short palindromic repeats
SAGE: Serial analysis of gene expression
MDR: Multiple drug resistance
NSG: NOD-SCID IL2rg null
OTP: Orthotopic
SQ: Subcutaneous
IP: Intraperitoneal
SRC: Subrenal capsule
MFP: Mammary fat pat
CNV: Copy number variation
MSI: Microsatellite instability
STR: Short tandem repeat
CDX: Cell line-derived xenograft
TIL: Tumor-infiltrating lymphocyte
DDR: DNA damage response
FDA: Food and drug administration (USA)
ALI: Air-liquid interface
TCR: T cell receptor
ICB: Immune checkpoint blockade
TME: Tumor microenviorment
CAF: Cancer-associated fibroblasts
CAM: Cancer-associated mesothelial
FTE: Fallopian tube epithelial
MISRII: Müllerian inhibitory substance type II receptor gene
